# Comment on Huang, X., et al. “Sourdough Fermentation Degrades Wheat Alpha-Amylase/Trypsin Inhibitor (ATI) and Reduces Pro-Inflammatory Activity”. *Foods* 2020, *9*, 943.

**DOI:** 10.3390/foods9101404

**Published:** 2020-10-03

**Authors:** Reijo Laatikainen

**Affiliations:** 1 Faculty of Medicine, Pharmacology, Medical Nutrition Physiology and Human Microbe Research Program, University of Helsinki, P.O. Box 63, FI-00014 Helsinki, Finland; reijo.laatikainen@booston.fi; 2 Booston Oy Ltd., Viikinkaari 6, FI-00790 Helsinki, Finland

I read with great interest the article titled “Sourdough Fermentation Degrades Wheat Alpha-Amylase/Trypsin Inhibitor (ATI) and Reduces Pro-Inflammatory Activity” by Huang et al. [1] in *Foods*. The authors demonstrated in vitro that the sourdough method has the potential to reduce potential negative ATI-related effects of wheat. These in vitro findings are of great academic interest, but robust randomized intervention trials are still lacking. 

As far as I know, the only clinical study addressing the role of ATIs directly in the human body is our pilot study [2] mentioned in the discussion and text [1]. In that study, ATI-reduced sourdough bread (139 g/24 h) and yeast bread (136 g/24 h) yielded similar inflammatory responses in sensitive population (IBS); no difference was found in IL-6, IL-8, or LBP. As a lead author of the paper [2], I want to draw attention to the confusing claim made by the authors in regard to our study [1]. The authors wrote: “In IBS patients, effects of ATI degradation are confounded by reduced levels of fermentable oligo-, di-, mono-saccharides, and polyols (FODMAPs) in sourdough bread. FODMAPs contribute to adverse intestinal symptoms in a majority of IBS patients, and particularly, the study of Laatikainen et al. [2] used bread where FODMAPs were strongly reduced in comparison to the control.” By writing so, the authors seem to confusingly suggest that the difference in the FODMAPs intake between the sourdough and yeast wheat bread periods had been a major confounder in our study and, as such, masked the effect of ATIs. I firmly disagree. 

The true situation is in conflict with what the authors may think. Sourdough baking reduces both FODMAP and ATI content of breads as the authors are aware of. Therefore, if anything, the minimal reduction of FODMAPs in sourdough wheat bread [1] overstate theoretical beneficial effects of ATI reduction (in sourdough bread). Reduction in ATIs and FODMAPs are both theoretically beneficial; they both might reduce gastrointestinal symptoms. In other words, the reduction of FODMAPs in our sourdough bread was not masking the effects ATI reduction—it made the low ATI bread look even better—still, in our study, the sourdough bread was not better tolerated than high-ATI bread (yeast bread). 

There also seem to be a misconception what constitutes clinically meaningful reduction in FODMAPs. The reduction in FODMAPs was not strong (“FODMAPs were strongly reduced”) as the authors wrote. In the mentioned study by our group, the observed difference in FODMAP intake was just 0.23 g/24 h (0.08 g vs. 0.31 g), i.e., difference in fructan content of the sourdough and yeast breads [1]. This absolute difference of 0.23 grams/24 h is a tiny difference and thus cannot be a major confounder in our study. It is very likely that this small difference of 0.23 g/24 h is not even a modest confounder, because the threshold for symptom triggering is considered to be per one eating occasion (usually 1 h) is 0.3 g of grain fructans [3]. Another randomized study showed that 1.6 grams of fructans/24 h, i.e., a more than six times bigger dose than 0.23 g, is likely to cause only modest symptom aggravation in some individual symptoms. Typically, a full-scale low-FODMAP diet reduces FODMAP intake by as much as 40 g/day [4], i.e., a more than 150 times bigger dose than 0.23 g. Taken together, from the dietetic and realistic perspective, the 0.23 g of fructans/day cannot be seen as a real confounder; it is far from clinically significant. The modest effects of fructans can possibly be seen in very sensitive populations if the doses are between 1–2 g/day at minimum. 

Taken together, our first clinical study on ATI was not significantly confounded by the minimally different FODMAP content of the breads, as the authors [1] seem to suggest, and our results make it very difficult to believe that ATI would play a major role in non-celiac gluten sensitivity (NCGS) or IBS. 

Another interesting and less-discussed dimension in regard to ATI research is the fact that gluten preparations have high ATI content and activity, as the authors have themselves previously described [5]. As the authors state in the text, placebo-controlled gluten studies have not been able to show a clear symptomatic effect of gluten preparations in functional gastrointestinal disorders or NCGS—but fructans cause symptom aggravation even when compared to gluten [6]. These findings challenge the view that ATI, as a natural co-passenger in gluten preparations, would have any significant symptomatic gastrointestinal effect in humans. 

Nonetheless, I eagerly wait for well-controlled randomized studies on the role of ATIs. The story will continue. 
